# Setting up for success: the effectiveness of telling children about their HIV status

**DOI:** 10.1002/jia2.70072

**Published:** 2025-12-23

**Authors:** Sarah Bernays, Ann Sellberg, Joni Lariat, Nicola Willis

**Affiliations:** ^1^ University of Sydney Camperdown New South Wales Australia; ^2^ London School of Hygiene and Tropical Medicine London UK; ^3^ Zvandiri Harare Zimbabwe

1

Once children and adolescents living with HIV (CALHIV) are on HIV treatment, telling them about their HIV status (also known as disclosure) is a core proactive and protective act. Supporting their understanding of HIV helps them recognize that the condition is manageable and untransmittable with effective treatment, which equips them to feel safe and minimize its impact on their lives [1, 2]. Evidence indicates that this is catalytic in achieving sustained viral suppression [3] and buoyant mental health [4]. This underpins the global recommendation that CALHIV be informed of their HIV status, explicitly naming it as such, once they reach school age (6–12 years, depending on cognitive skills and social maturity) [5].

Despite this, estimates indicate that around 40% of adolescents living with HIV (ALHIV), 13 years and older, have not been told about their HIV status [3]. The absence of clinical documentation limits the clarity of our understanding but also signifies our persistent under‐valuing of its impact on ALHIVs’ health outcomes. Ironically, if telling a child about their HIV status (disclosing) was classified as a clinical rather than a psychosocial intervention, we would more likely recognize it as a transformative intervention worthy of investment.

Conversations to tell CALHIV about their HIV status are hard. For caregivers, who are generally designated this responsibility, this often involves sharing information about the HIV status of biological parents. The anticipated emotional weight of these discussions means that they are commonly clouded in obfuscation and deferral. Meanwhile, CALHIV do not know the truth about the nature of their condition or why they are taking treatment. These delays have profound sequelae: deciphering their HIV status on their own, sometimes late into their adolescence, can provoke acute anxiety, distrust and moral injury, which can, in turn, reflect in poor mental health and suboptimal viral suppression rates [6].

Our stuttering implementation of telling children about their HIV status over the last few decades has not been because we do not know why or how to do it well. Best practices are well‐elucidated [7]: disclosure is a process, not a single event [8], requiring ongoing, age‐appropriate conversations that help CALHIV understand the implications and manageability of their HIV status safely and progressively. The recently published WHO guidance reinforces the need to act on proven approaches [7]. It identifies 25 interventions that effectively support HIV status disclosure among CALHIV, built around core elements such as respect for autonomy and dignity, tools that enhance engagement, and attention to health and environmental contexts. Collectively, these components improve confidence, acceptance, communication and knowledge, while providing essential support for disclosure decisions.

Three key challenges hinder telling CALHIV their HIV status in a timely and supported manner: (1) limited time and capacity for ongoing counselling once discussions begin [9]; (2) caregivers’ concerns about initiating the process [10]; and (3) uncertainty about a child's existing awareness [11]. Despite the escalating pressures caused by reduced funding, these challenges can be overcome and multiple positive effects realized by integrating the peer counselling cadre and the simple, streamlined and systematic tracking of a child's “disclosure” status to strengthen existing clinical care.

**Peer counsellors, who have the time and skills, can work alongside healthcare providers and caregivers to facilitate supportive conversations**: The pressures on healthcare providers in busy clinics have long clashed with the time needed to support a child to understand the implications of their HIV status [12]. Peer counsellors mitigate this by supporting ongoing conversations which foster CALHIV's emerging HIV literacy and agency to effectively manage (and minimize) the implications of their HIV status [12, 13]. The feasibility of achieving near‐universal, supported telling has been demonstrated through the integration of a peer‐support model (Zvandiri) in Zimbabwe, South Africa and South Sudan [14]. In this approach, peer counsellors collaborate with healthcare providers in clinics and communities to update “disclosure status” in clinical records, provide ongoing counselling and connect CALHIV safely with peers, leading to dramatic improvements in “disclosure” rates [13, 14].

The process of telling a child about their HIV status works best when healthcare providers and peer counsellors can support caregiver‐led conversations. Evidence indicates that caregivers’ reticence to tell their children dissipates if they receive ongoing guidance from peers and providers. By demonstrating the undiminished potential of someone growing up with HIV, through their embodiment of health and capability, peer counsellors prompt caregivers to recalibrate their understanding of how HIV will affect their child's future, which eases their concerns about telling their child about their HIV status [15]. Zvandiri recently developed “Walking in my shoes” (WIMS), an interactive tool that helps caregivers engage in meaningful conversations and provide effective care, particularly about HIV status [15]. WIMS includes three animated scenarios on disclosure, stigma and adherence, each with discussion questions that encourage caregivers to “stand in the shoes” of the young person, shift their perspective, and with that, strengthen the care they provide. Preliminary data show promising results.

**Adopting a universal “Telling about HIV” symbol for tracking and monitoring is a feasible and effective mechanism to ensure a shared understanding of current progress in telling a child/young person about their HIV status**: Despite its clinical relevance, healthcare workers often do not know whether the CALHIV in their care are aware of their HIV status [6]. Such uncertainty can lead to deferring conversations, delaying access to support that relies on the child knowing their HIV status.

To address this, Medicine Sans Frontier (MSF) has implemented a simple system of recording a symbol in a child/young person's file: an empty circle is drawn when there has been no form of disclosure; this circle is half‐shaded for partial disclosure (told about the virus, immune system and medicines without naming HIV) and fully shaded when full disclosure has occurred. MSF has been advocating for this denotation through health centre mentorship visits, supported by a flipchart tool with pictures to support the counsellor in providing both partial and full disclosure [16]. This approach has been adopted by the Zimbabwe Ministry of Health and Child Care in the revised Operational Standard Delivery Manual, with healthcare workers collaborating closely with peer counsellors. This is supported by a checklist tool developed to support CALHIV to transition to adult care, including a section on HIV and Anti‐Retroviral treatment (ART) literacy. Used together, this has strengthened documentation and linkages of CALHIV to “disclosure” services, and improved disclosure rates [17].

Galvanized by support from peer counsellors and a universal progress‐tracking symbol system, the skilful, timely and safe telling of every child about their HIV status is feasible. This requires (1) simple scaffolded steps built into clinical care; (2) healthcare workforce competencies to support delivery; and (3) indicators with monitored targets to drive uptake and sustain momentum. Despite resource pressures, ensuring CALHIV are told about their HIV status sets them up for success and will have a catalytic effect on all their health outcomes.

## COMPETING INTERESTS

The authors have no conflicts of interest to report.

## AUTHORS’ CONTRIBUTIONS

SB: Conceptualization, writing—original draft, writing—reviewing and editing. AS: Conceptualization, writing—original draft, writing—reviewing and editing. JL: Writing—reviewing and editing. NW: Conceptualization, writing—reviewing and editing. All authors have read and approved the final manuscript.

## Data Availability

Data sharing is not applicable to this article as no datasets were generated or analysed which are drawn on for this Viewpoint. All references cited are in the public domain.
